# Identification of *Candida parapsilosis* *sensu lato* and antifungal susceptibility testing of candidemia patients in a tertiary-care hospital in Malaysia

**DOI:** 10.1128/spectrum.01457-25

**Published:** 2025-11-17

**Authors:** Dina Yamin, Nik Mohd Noor Nik Zuraina, Azian Harun, Nur Waliyuddin Hanis Zainal Abidin, Abubakar Muhammad Wakil

**Affiliations:** 1 Department of Medical Laboratory Sciences, School of Science, The University of Jordan 54658, Amman, Jordan; 2 Department of Medical Microbiology and Parasitology, School of Medical Sciences, Universiti Sains Malaysia 596158 , Kubang Kerian, Kelantan, Malaysia; 3 Department of Veterinary Clinical Studies, Faculty of Veterinary Medicine, Universiti Malaysia Kelantan 356743 , Kota Bharu, Kelantan, Malaysia; 4 Hospital Universiti Sains Malaysia 69847, Kota Bharu, Kelantan, Malaysia; 5 Human Identification/DNA Unit, School of Health Sciences, Universiti Sains Malaysia 26689, Kubang Kerian, Kelantan, Malaysia; 6 Department of Veterinary Physiology and Biochemistry, Faculty of Veterinary Medicine, University of Maiduguri Maiduguri, Maiduguri, Borno State, Nigeria; Peking University People's Hospital, Beijing, China

**Keywords:** *Candida parapsilosis sensu stricto*, PCR-ITS, E-test, antifungal drugs, blood culture

## Abstract

Candidemia is the fourth most common nosocomial bloodstream infection and the most prevalent nosocomial fungal infection. *Candida parapsilosis* complex is the second most frequently identified species from blood samples in different countries worldwide, exceeding the prevalence of *Candida albicans* in some hospitals. Proper conventional identification of *C. parapsilosis* to subspecies level and confirming this identification using molecular methods such as amplifying and sequencing the internal transcribed spacer–polymerase chain reaction (ITS-PCR) help promptly in final diagnosis of candidemia. In addition, antifungal susceptibility testing (AFST) is becoming mandatory for each individualized sample; a reliable estimation of antifungal susceptibility of *Candida* causing bloodstream infections is increasingly important to track changes in the antifungal sensitivity/resistance of *Candida* species and define the burden of antifungal-resistant strains. Here, molecular research was conducted to identify the subspecies of *C. parapsilosis* and identify patterns of antifungal sensitivity/resistance in a tertiary-care hospital in Malaysia. This study involved a total of 50 *C*. *parapsilosis* complex isolates recovered from catheters and blood cultures of patients. Subspecies identification was conducted by conventional methods and confirmed by ITS-PCR, a molecular-based technique. Antifungal susceptibility to azoles, amphotericin B, and echinocandins was tested by E-test method, and results were interpreted according to Clinical and Laboratory Standards Institute clinical breakpoints. Three different subspecies of *C. parapsilosis* complex were identified: 40 *C. parapsilosis sensu stricto*, 9 *Candida orthopsilosis,* and 1 *Candida metapsilosis*. Most of the strains were susceptible to all antifungals studied, with only a few resistant cases observed. However, despite the limited number of resistant isolates, this study highlights the continuous need to conduct species identification and AFST studies to assess and monitor the growing burden of candidemia, control the effect of potential antifungal resistance, and implement regional surveillance programs to prevent further rise in candidemia rates. Therefore, it is of substantial significance to carry out such types of studies in different countries all over the world to understand the great burden of candidemia and emergence of resistant strains. Early diagnosis of fungal infections in general and candidemia specifically is of essential importance for the proper treatment, prevention, and management of nosocomial fungal infection, such as catheter-related bloodstream infections.

**IMPORTANCE:**

*Candida parapsilosis* is an emerging cause of bloodstream infections in hospitals, particularly in vulnerable patients using catheters or medical devices. These infections are serious and can be difficult to treat, especially if the yeast becomes resistant to common antifungal medications. In this study, we examined *Candida* samples from a Malaysian hospital to identify the specific types of *C. parapsilosis* and test their resistance to antifungal drugs. We found that most strains were still sensitive to treatment, but some showed early signs of resistance, especially to itraconazole. This work highlights the urgent need for hospitals to regularly monitor fungal infections and adjust treatment plans based on up-to-date lab testing. Understanding which strains are present and how they respond to drugs helps doctors choose the most effective treatment, improves patient outcomes, and supports infection control efforts to prevent future outbreaks of resistant fungal infections.

## INTRODUCTION

Candidemia is the fourth most prevalent nosocomial bloodstream infection and the most frequent nosocomial fungal infection (1–4). *Candida parapsilosis sensu lato* is the second most frequently isolated species from blood in Asia, Latin America, and some European countries, surpassing the *Candida albicans* frequency in some hospitals (5–9). In Malaysia, the rate of *C. albicans* infections was still found to be dominant compared to other species in most studies. However, there have been several reports of non-albicans *Candida* (NAC) marginally overtaking *C. albicans* as the leading cause of bloodstream infections (10). In Malaysia, the epidemiology of candidemia mirrors global trends, with an increasing proportion of NAC species reported in tertiary care settings. Recent studies have documented that *C. parapsilosis* is now among the most frequently isolated species from blood cultures, sometimes surpassing *C. albicans* in prevalence (10). For instance, a recent report highlighted that *C. parapsilosis* was noted to be the most predominant species causing candidemia (11). *C. parapsilosis sensu lato* includes three closely related species: *C. parapsilosis sensu stricto*, *Candida orthopsilosis*, and *Candida metapsilosis* (12).

The escalating resistance of *Candida* species to antifungal therapy presents a growing concern within the clinical management of fungal infections. This issue is particularly pressing given the rising incidence of candidemia and other invasive candidiasis, notably in immunocompromised populations (13). Effective control remains hindered by several systemic obstacles, including limited access to advanced diagnostics, suboptimal infection prevention practices, and persistent gaps in the clinical understanding of fungal pathogens (14). Compounding the problem is the frequent reliance on empirical antifungal treatment without microbiological confirmation, which has contributed to the selection and proliferation of resistant strains. The emergence of multidrug-resistant species such as *Candida auris*—known for their diagnostic complexity, therapeutic challenges, and capacity for nosocomial transmission—further complicates the landscape (14). These challenges collectively highlight the critical need for improved diagnostic infrastructure, robust infection control measures, antifungal stewardship programs, and continued professional training to enhance the management of drug-resistant *Candida* infections.

Despite the increasing prevalence of various *Candida* species, with over 700,000 cases of invasive candidiasis being reported globally each year (15), there are still limited data in Malaysia on the epidemiology and antifungal susceptibility of *Candida* species infections, especially on *C. parapsilosis sensu lato*. In Malaysia, available data remain fragmented and are mostly derived from multicenter surveillance or general reports that do not distinguish between species complexes. To date, there is limited institution-specific evidence from tertiary referral hospitals, which are key sites for managing high-risk patients such as those with hematological malignancies, solid organ transplants, prolonged intensive care unit (ICU) admissions, and indwelling medical devices. Our hospital, being one of the largest tertiary-care centers in the region, caters to a broad catchment area and regularly manages patients with these risk factors. Recent internal surveillance reports have indicated an increasing number of bloodstream infections caused by NAC, with *C. parapsilosis* frequently suspected based on preliminary phenotypic identification. This local trend raises clinical concern given the species’ association with catheter-related infections, biofilm formation, and variable antifungal susceptibility profiles. Therefore, a focused investigation at this institution is both epidemiologically relevant and clinically necessary to guide local treatment policies, strengthen infection control practices, and provide baseline data for future multicenter comparisons within Malaysia.

In order to effectively control *C. parapsilosis* infections, it is important to determine the prevalence and antifungal susceptibility pattern of this *Candida* species infections in all geographical regions of Malaysia. The aims of this study were to determine the prevalence of the three strains of the *C. parapsilosis* species complex as a candidemia agent in hospitalized patients from the tertiary hospital in Malaysia—specifically, to quantify the proportion of *C. parapsilosis sensu stricto*, *C. orthopsilosis*, and *C. metapsilosis* among candidemia cases—and to evaluate its antifungal susceptibility profiles.

This study was deliberately designed as a single-center, laboratory-based investigation, focusing specifically on the clinical microbiology and antifungal susceptibility patterns of *C. parapsilosis sensu lato*. The research design reflects our aim to generate detailed and high-quality microbiological data within the practical constraints of our institutional resources and available timeframe.

## MATERIALS AND METHODS

### Isolate collection

The study was conducted in accordance with the guidelines of the Declaration of Helsinki and was approved by the Human Research Ethics Committee of Universiti Sains Malaysia (JEPeM-USM-16040162). Between 2017 and 2019, all 50 available isolates of *C. parapsilosis sensu lato* from 27 patients admitted to Hospital Universiti Sains Malaysia (HUSM), Malaysia, were included in this study. Each patient had at least one blood culture positive for candidemia, confirmed by conventional species identification from peripheral or central blood samples processed by the Laboratory of Microbiology and Parasitology. For patients with indwelling catheters, both peripheral and central blood cultures were collected, resulting in multiple isolates from the same patient in some cases; however, only the first isolate per patient was included in the antifungal susceptibility testing (AFST) to represent that patient’s infection. In total, 29 isolates were recovered from peripheral blood and 21 from central catheter blood. All isolates underwent molecular species identification through amplification and sequencing of the internal transcribed spacer (ITS) region at the Department of Medical Microbiology and Parasitology, USM, and were recorded in the Laboratory Information System.

### Species identification

The isolates were initially identified as *C. parapsilosis sensu lato* using the commercial biochemical identification kits such as API 20C AUX, ID 32C, or Vitek (Bio-Mérieux, Marcy l’Etoile, France), all of which are widely used for phenotypic identification of yeasts based on carbon assimilation profiles. These isolates were collected between 2017 and 2019 as part of routine diagnostic procedures, which included both species identification and AFST. For the purpose of this research, molecular identification was subsequently performed using polymerase chain reaction amplification and sequencing of the ITS (16). DNA extraction was performed following the previously published protocol (17).

### Antifungal susceptibility testing

Only the first isolate from each patient was included in the AFST; repeat isolates from the same patient were excluded to avoid duplication. Each isolate was tested once, with no technical or biological replicates, as the study focused on representative susceptibility testing of clinical samples. Antifungal susceptibility to azoles, amphotericin B (AMB), and echinocandins was tested by a gradient diffusion method commercially named E-test (bioMérieux, France). The *in vitro* antifungal activity of fluconazole (FLC), voriconazole (VRC), and posaconazole (PSC) (Pfizer, USA); caspofungin (CSF) (Merck & Co., USA); and itraconazole (ITC) and AMB (Sigma-Aldrich) was evaluated according to Clinical and Laboratory Standards Institute (CLSI) document (M27-S4, 4th edition, 2012) that was current at the time of testing, which represented the most up-to-date guideline available during the study period as shown in Table S1 (18–20). However, updated breakpoints are available in the more recent CLSI M60 document (3rd edition, 2023), which was released after completion of this study. The antifungals selected for testing reflect those most commonly prescribed in our hospital for the management of candidemia and other invasive fungal infections, with FLC, VRC, CSF, and AMB representing the frontline therapeutic options, while ITC and PSC are used as alternatives in selected cases.

## RESULTS

A total of 50 *C*. *parapsilosis sensu lato* were identified conventionally. Of which, 40 (80%) isolates were molecularly identified as *C. parapsilosis sensu stricto*, nine (18%) were *C. orthopsilosis* (between the orthopedic ward, emergency department, pediatric oncology, and general surgery), and only one (2%) isolate was *C. metapsilosis* according to ITS region, and their ITS sequences were deposited in GenBank database in NCBI under accession numbers (OP618172–OP618210). Their detailed characteristics are demonstrated in Table 1. It is worth mentioning that the patients with *C. parapsilosis* generally had catheters.

**TABLE 1:** Detailed characteristics of *C. parapsilosis* isolated from blood samples^*i*^

Sample ID	Gender	Age	Sample source	Specimen date	Unit/ward description	Species^*h*^
USM07	Female	76 years	C	31/03/2017	Female medical	*C. parapsilosis*
**USM01**	Male	72 years	P	24/06/2017	Emergency department	*C. parapsilosis*
**USM02**	Male	60 years	P	25/06/2017	General surgery	*C. parapsilosis*
**USM05*^a^***	Female	49 years	C	29/08/2017	HDU surgical	*C. parapsilosis*
USM08^***a***^	Female	49 years	C	29/08/2017	HDU surgical	*C. parapsilosis*
**USM11**	Male	34 years	C	22/01/2018	Oncology	*C. parapsilosis*
**USM06** ^*b*^	Female	12 years	P	30/03/2018	Pediatric oncology	*C. parapsilosis*
USM09^*b*^	Female	12 years	P	08/04/2018	Pediatric oncology	*C. parapsilosis*
USM10	Male	40 years	C	21/05/2018	CCU	*C. parapsilosis*
USM13	Male	63 years	P	02/07/2018	Medical	*C. parapsilosis*
USM14	Male	63 years	C	04/07/2018	Medical	*C. parapsilosis*
**USM03**	Female	37 years	P	04/08/2018	Surgical ICU	*C. parapsilosis*
USM18^*c*^	Female	54 years	P	09/12/2018	Surgical HDU	*C. parapsilosis*
USM19^*c*^	Female	54 years	P	06/12/2018	Surgical HDU	*C. parapsilosis*
**USM21**	Female	54 years	P	22/01/2019	Emergency department	*C. parapsilosis*
**USM22^*d*^**	Male	3 months	P	24/01/2019	Neonatal ICU	*C. parapsilosis*
USM27^***d***^	Male	3 months	P	28/01/2019	Neonatal ICU	*C. parapsilosis*
USM23^*e*^	Male	10 years	C	24/01/2019	Pediatric oncology	*C. parapsilosis*
USM25^*e*^	Male	10 years	C	28/01/2019	Pediatric oncology	*C. parapsilosis*
USM26^*e*^	Male	10 years	P	28/01/2019	Pediatric oncology	*C. parapsilosis*
USM28^*e*^	Male	10 years	P	03/02/2019	Pediatric oncology	*C. parapsilosis*
**USM29** ^*e*^	Male	10 years	C	07/02/2019	Pediatric oncology	*C. parapsilosis*
**USM24**	Female	75 years	P	27/01/2019	Emergency department	*C. parapsilosis*
**USM30**	Male	18 days	P	28/03/2019	Neonatal ICU	*C. parapsilosis*
**USM32**	Male	5 years	C	31/03/2019	Pediatric oncology	*C. parapsilosis*
**USM34**	Female	72 years	P	27/06/2019	Emergency department	*C. parapsilosis*
**USM36** ^*f*^	Male	6 years	P	03/08/2019	Pediatric	*C. parapsilosis*
USM37^*f*^	Male	6 years	C	23/08/2019	Pediatric	*C. parapsilosis*
USM39*^f^*	Male	6 years	P	23/08/2019	Pediatric	*C. parapsilosis*
USM40^*f*^	Male	6 years	P	24/08/2019	Pediatric	*C. parapsilosis*
USM41*^f^*	Male	6 years	P	29/08/2019	Pediatric	*C. parapsilosis*
USM42^*f*^	Male	6 years	C	29/08/2019	Pediatric	*C. parapsilosis*
**USM38**	Male	2 years	P	22/08/2019	Surgical	*C. parapsilosis*
**USM43** ^*g*^	Female	5 years	P	11/09/2019	Pediatric oncology	*C. parapsilosis*
USM44^*g*^	Female	5 years	C	11/09/2019	Pediatric oncology	*C. parapsilosis*
USM45^*g*^	Female	5 years	C	13/09/2019	Pediatric oncology	*C. parapsilosis*
USM46^*g*^	Female	5 years	C	15/09/2019	Pediatric oncology	*C. parapsilosis*
USM47^*g*^	Female	5 years	C	15/09/2019	Pediatric oncology	*C. parapsilosis*
USM48^*g*^	Female	5 years	C	19/09/2019	Pediatric oncology	*C. parapsilosis*
**USM50**	Female	62 years	P	15/10/2019	Surgical ICU	*C. parapsilosis*
USM12	Male	78 years	P	25/01/2018	Emergency department	*C. orthopsilosis*
USM15	Female	81 years	P	29/08/2018	Emergency department	*C. orthopsilosis*
USM31	Male	5 years	C	29/03/2019	Pediatric oncology	*C. orthopsilosis*
USM49	Male	21 years	P	10/09/2019	General surgery	*C. orthopsilosis*
USM51	Male	64 years	P	11/12/2019	Orthopedic	*C. orthopsilosis*
USM52	Male	64 years	C	17/11/2019	NA	*C. orthopsilosis*
USM53	Male	64 years	C	19/11/2019	Orthopedic	*C. orthopsilosis*
USM54	Male	64 years	C	19/11/2019	Orthopedic	*C. orthopsilosis*
USM55	Male	64 years	P	19/11/2019	Orthopedic	*C. orthopsilosis*
USM35	Male	54 years	P	29/06/2019	NA	*C. metapsilosis*

^*a*^ Isolates were collected from the same patient (Patient a).

^*b*^ Isolates were collected from the same patient (Patient b).

^*c*^ Isolates were collected from the same patient (Patient c).

^*d*^ Isolates were collected from the same patient (Patient d).

^*e*^ Isolates were collected from the same patient (Patient e).

^*f*^ Isolates were collected from the same patient (Patient f).

^*g*^ Isolates were collected from the same patient (Patient g)*.*

^*h*^ Species identity was determined both conventionally and molecularly according to ITS sequences. USM isolates shown in bold were tested for AFST. The isolates not in bold were not tested either due to unavailability of the testing kit, because they were replicates of another tested isolate, or because they were not identified as *C. parapsilosis*.

^*i*^ P, peripheral blood; C, central blood withdrawn from catheters; ICU, intensive care unit; CCU, coronary care unit; HDU, high dependency unit, and NA, not available.

The AFST results of the *C. parapsilosis sensu stricto* strains according to CLSI M27-S4 are presented in Table 2, and their frequencies are presented in Table 3. Resistance occurs when a fungal organism can survive and grow even in the presence of an antifungal drug at concentrations that would normally inhibit or kill it. For AMB, *C. parapsilosis sensu stricto* isolates showed a narrow range of minimal inhibitory concentration (MIC) values (MIC50, 0.5 µg/mL; range, 0.047 to 2.0 µg/mL) and an MIC90 of 1 µg/mL, with only one strain showing resistance to AMB with the MIC values of 2.0 µg/mL (Table 4). Azoles and echinocandins showed good activity. The majority of the strains showed a low FLC MIC, and only one strain (6%) had the MIC values that were considered susceptible dose dependent (SDD). All the strains were susceptible to VRC and PSC (range, 0.012 to 0.12 µg/mL) while showing some resistance to ITC (MIC50, 0.12 µg/mL; range, 0.06 to 0.38 µg/mL), where three strains were SDD and two strains were resistant to ITC. For the echinocandins, only CSF was tested. However, all the strains were considered susceptible to CSF (Table 3). No resistant strain was identified against CSF (MIC50, 1.0 µg/mL; range, 0.25 to 2.0 µg/mL) as shown in Table 4.

**TABLE 2:** The AFST results of the *C. parapsilosis* strains according to CLSI M27-S4^*a*^

Sample	AMB	FLC	VRC	CSF	PSC	ITC
USM01	S	S	S	S	S	S
USM02	S	S	S	S	S	S
USM03	S	S	S	S	S	SDD
USM05	S	S	S	S	S	S
USM06	S	S	S	S	S	SDD
USM11	S	S	S	S	S	R
USM21	S	S	S	S	S	SDD
USM22	S	S	S	S	S	S
USM24	S	S	S	S	S	S
USM29	S	SDD	S	S	S	R
USM30	S	S	S	S	S	S
USM32	S	S	S	S	S	S
USM34	R	S	S	S	S	NA
USM36	S	S	S	S	S	S
USM38	S	S	NA	S	S	NA
USM43	S	S	S	S	S	NA
USM50	S	S	S	S	NA	NA

^*a*^ AMB, amphotericin B; FLC, fluconazole; VRC, voriconazole; CSF, caspofungin; PSC, posaconazole; ITC, itraconazole; S, susceptible; SSD, susceptible dose dependent; R, resistant; NA, not available.

**TABLE 3:** Percentages of susceptible and resistant *C. parapsilosis* isolated from blood culture according to CLSI M27-S4^*a*^

	No. (%) based on M27-S4		
Antifungal drug	S (%)	SDD/I (%)	R (%)
Amphotericin B	16 (94)		1 (6)
Fluconazole	16 (94)	1 (6)	
Itraconazole	8 (62)	3 (23)	2 (15)
Posaconazole	16 (100)		
Voriconazole	16 (100)		
Caspofungin	17 (100)		

^*a*^ S, susceptible; SSD/I, susceptible dose dependent/intermediate; R, resistant.

**TABLE 4:** MIC distribution of *C. parapsilosis* isolates analyzed in this study^*a*^^,^^*b*^

Antifungal drug	MIC range (µg/mL)	MIC 50 (µg/mL)	MIC 90 (µg/mL)
Amphotericin B	0.047–2.0	0.5	1.0
Fluconazole	0.5–4.0	1.0	2.0
Itraconazole	0.06–0.38	0.12	0.38
Posaconazole	0.012–0.12	0.06	0.12
Voriconazole	0.012–0.12	0.03	0.12
Caspofungin	0.25–2.0	1.0	2.0

^*a*^ MIC was determined by the gradient tests.

^*b*^ MIC, minimal inhibitory concentration.

Unfortunately, we could not compare the AFST of the three *C. parapsilosis* species since no AFST results were reported for the less pathogenic *Candida* species, namely, *C. orthopsilosis* and *C. metapsilosis* strains at that time.

## DISCUSSION

Invasive candidiasis caused by NAC species has increased significantly in recent years. Continuous epidemiological surveillance of *Candida* species-caused invasive fungal infections and their antifungal susceptibility profiles is required to monitor changes and provide up-to-date information for accurate therapy and proper candidiasis management. In this 2-year period of study sampling, a total of 50 *C*. *parapsilosis* complexes were accounted for among the prominent NAC species isolated from patients with candidemia. Similar to other studies, *C. parapsilosis sensu stricto* has predominated the species, followed by a small number of *C. orthopsilosis* and *C. metapsilosis. C. parapsilosis* has become an increasingly important cause of bloodstream infections among NAC isolates worldwide, with notable regional variations. In Malaysia, a retrospective study conducted at HUSM found that *C. parapsilosis* was the most common species in candidemia, accounting for 29.2% of cases from 2001 to 2018, surpassing *C. albicans* (20.1%) and *C. tropicalis* (18.7%) as the leading cause of nosocomial candidemia (11). This trend is consistent with findings from China (2005–2020), where *C. parapsilosis* has also emerged as a predominant NAC species in bloodstream infections, representing 86.3% of NAC (21). These data highlight the shifting landscape of candidemia, with *C. parapsilosis* playing an increasingly prominent role in both Malaysia and other parts of Asia, underscoring the need for ongoing surveillance and targeted infection control efforts.

It has been reported that an evident progressive shift of *C. albicans* toward NAC species is being witnessed worldwide (22). Species distribution varies according to the geographical region, the variability in the use of antifungal drugs and medical devices in the candidemia treatment and therapy, and the age and comorbidities of the patients. For instance, an increasing rate of *Candida glabrata* infections has been reported in Northern Europe and the United States, and it prevails in patients aged above 60 years. However, *C. parapsilosis* currently emerges in Asia, Southern Europe, and Latin America, and it is prominent in newborns (22). In Malaysia, previous data on the distribution of *Candida* species isolated from patients’ blood samples over an 18-year period (2001–2018) showed that NAC species accounted for 79.9% of total *Candida* isolates, with the most common species being *C. parapsilosis* (11). Other studies conducted in Malaysia also reported the higher rate of *C. parapsilosis* candidemia infections over *C. albicans* (23, 24).

A number of risk factors, including the organism’s ability to grow in parenteral nutrition solutions and its endowment for intravascular and prosthetic devices, could explain the rise in *C. parapsilosis* complex infections (25). Furthermore, immunocompromised individuals, patients requiring a central venous catheter or indwelling device for an extended period of time, and those undergoing gastrointestinal surgery are at high risk of infection with *C. parapsilosis* (26). As for this study, most of the isolates were derived from the oncological and surgical wards. Patients with indwelling catheters were found to be significantly more likely to have *C. parapsilosis* infection, regardless of ward (80.9%, *n* = 17/21). Meanwhile, all the patients from the orthopedic wards were positive for *C. orthopsilosis* (100%, *n* = 4/4).

Recently, the global prevalence of antifungal-resistant *C. parapsilosis* has been systematically reviewed (27). Among antifungal drugs, azoles (especially FLC) and AMB have been the most commonly used to treat invasive candidiasis. The widespread use of FLC for both prophylactic and empirical therapy has been attributed to a shift in the epidemiology of *Candida* infections. Patients who are receiving or have recently received azole antifungal therapy are more likely to get NAC infections. Resistance to azole drugs has previously been reported in *C. parapsilosis* isolates, particularly in invasive *Candida* infections (5, 28, 29). The evaluation of antifungal activities of *C. parapsilosis* against FLC, VRC, PSC, and CSF in this study showed good susceptibility to these four drugs, with the MIC ranging from 0.012 µg/mL for VRC and PSC to 4.0 µg/mL for FLC. Exceptionally, resistance was observed in ITC and AMB, by which 38% (*n* = 5/13) and 6% (*n* = 1/16) of the *C. parapsilosis* isolates showed resistant or SDD to azole drug ITC and AMB, respectively.

Reduced susceptibility toward ITC was also reported by previous studies in Malaysia (20, 30), indicating that certain fungal isolates may exhibit decreased sensitivity to the drug while still responding under adjusted treatment conditions. An earlier report in 2005 discovered zero ITC resistance in *C. parapsilosis* isolated from candidemia cases, but 80% (*n* = 4/5) of the isolates exhibited an SDD pattern to ITC (30). Later in the year 2009, another study revealed the presence of ITC resistance and an SDD profile in 38% (*n* = 5/13) of the *C. parapsilosis* isolates (20). The reduced susceptibility to ITC could be attributed to the long-term use of this drug as empirical therapy for invasive candidiasis in Malaysian hospital settings. Although most of the studies reported low resistance rate (< 6%) of *C. parapsilosis* complex to azoles globally (21), higher prevalence of ITC and FLC resistance has also been reported in Iran (89%, *n* = 94/105) and South Africa (63%, *n* = 332/551), respectively (31, 32). The evidence of ITC resistance in *C. parapsilosis* isolates marks a considerable issue to look forward to.

Although resistance toward AMB was recorded in this study, most isolates showed good susceptibility to this antifungal drug. An average low prevalence of AMB-resistant *C. parapsilosis* (1.3%) has been reported worldwide, with some countries reporting a slightly higher resistance rate (27). The low incidence of AMB resistance among *C. parapsilosis* was also reported previously in Malaysia, with susceptibility rates of 95%–100% (20, 33). However, due to the small number of isolates used in this study, the low incidence of AMB resistance may not characterize the actual prevalence in the population. Hence, the significance of AMB resistance in clinical settings should be considered. Ongoing evidence-based surveillance is strongly advised to track changes in antifungal resistance patterns and species distribution within the *C. parapsilosis* complex.

The findings of this study have important clinical implications for the empirical management of candidemia in our institution. The predominance of *C. parapsilosis sensu stricto* (80%) suggests that empirical antifungal therapy in our setting should prioritize agents with proven activity against this species. The observed overall susceptibility to azoles, particularly FLC and VRC, supports their continued use as first-line options for non-critical patients, while echinocandins such as CSF remain suitable for severely ill or high-risk patients, given their 100% susceptibility in the tested isolates. The presence of isolated resistance to AMB and ITC, as well as a small proportion of FLC SDD strains, highlights the need for ongoing antifungal susceptibility surveillance and careful consideration of dose adjustments or alternative therapy when resistance is suspected. These data may inform local antifungal prescribing policies by emphasizing targeted empirical therapy based on prevailing susceptibility patterns, reinforcing the importance of species-level identification, and guiding stewardship interventions to minimize the emergence of resistance. Furthermore, the inability to perform susceptibility testing for *C. orthopsilosis* and *C. metapsilosis* underscores the need for expanded testing capabilities to fully capture the resistance landscape of the *C. parapsilosis* complex.

This current study is a single-center study involving a limited number of isolates collected over 2 years. Therefore, taking into account the limitations, a study with larger sample size, extended period of sampling, inclusive sociodemographic and epidemiological data of each patient, and further characterization of antifungal resistance pattern for each subspecies is recommended for continuous input toward better management of candidemia in the future. In line with these limitations, it is worth noting that in two cases, unit/ward information was unavailable due to incomplete documentation at the time of strain archiving. Notably, these included isolates of *C. orthopsilosis* and *C. metapsilosis*, which were rare in our collection. This highlights the importance of systematic and comprehensive metadata recording to ensure that the clinical context of such uncommon isolates can be accurately interpreted and effectively utilized in future research and clinical decision-making. An additional limitation of this study is the time gap between data collection (2017–2019) and publication. However, the findings still provide important baseline data on the local epidemiology and antifungal susceptibility patterns of *C. parapsilosis sensu lato*. Despite the delay, the study remains valuable as it offers critical insights into the epidemiology and resistance profiles of *C. parapsilosis* within a specific healthcare setting, serving as a valuable point of comparison for future research in the field.

### Conclusion

In this study, we noticed that *C. parapsilosis* showed low MICs to all the tested drugs. The discrimination of cryptic species within the *C. parapsilosis* species complex would not have a considerable clinical utility. However, surveillance is essential to study the epidemiology of this complex, determine changes in species distribution, and identify resistant strains, especially in candidemia patients whose treatment options are limited.

## Data Availability

Data are contained within the article or databases.

## SUPPLEMENTAL MATERIAL

Table S1 spectrum.01457-25-s0001.pdf Interpretive breakpoints for the reference methods for antifungal susceptibility testing of *C. parapsilosis*.

ASM does not own the copyrights to Supplemental Material that may be linked to, or accessed through, an article. The authors have granted ASM a non-exclusive, world-wide license to publish the Supplemental Material files. Please contact the corresponding author directly for reuse.
