# Supplementary material for: Identification of Candida parapsilosis sensu lato and antifungal susceptibility testing of candidemia patients in a tertiary-care hospital in Malaysia
Source: Microbiol Spectr. 2025 Nov 17;14(1):e01457-25. doi: 10.1128/spectrum.01457-25 (PMC12772269; doi:10.1128/spectrum.01457-25)
Supplement: Table S1 — Interpretive breakpoints for the reference methods for antifungal susceptibility testing of C. parapsilosis. [file spectrum.01457-25-s0001.pdf]

Table S1. Interpretive breakpoints for the reference methods for antifungal susceptibility testing of *C. parapsilosis.*

Antifungal	CLSI M27-A3 [1]			CLSI M27-S4 [2]		
	S	SDD/I	R	S	SDD/I	R
Amphotericin B*	≤1		>1	≤1		>1
Fluconazole	≤8	16-32	≥64	≤2	4	≥8
Itraconazole	≤0.125	0.25-0.5	≥1	≤0.125	0.25-0.5	≥1
Ketoconazole*	≤0.125		>0.125	≤0.125		>0.125
Posaconazole*	≤1		>1	≤0.125	0.25-0.5	≥1
Voriconazole	≤1	2	≥4	≤0.125	0.25-0.5	≥1
5-Flucytosine[#]	≤4	8-16	≥32	≤4	8-16	≥32
Caspofungin	≤2		>2	≤2	4	≥8

[1]Data compiled from reference (1). [2]Data compiled from reference (2). *The breakpoints of amphotericin B, ketoconazole and posaconazole were compiled from (3) since their breakpoints have not been established yet by CLSI. [#]5-Flucytosine breakpoint was compiled from (3) according to CLSI.

1. Arendrup MC. Candida and candidaemia. Susceptibility and epidemiology Dan Med J. 2013;60(11):B4698.
2. Pfaller M, Diekema D. Progress in antifungal susceptibility testing of Candida spp. by use of Clinical and Laboratory Standards Institute broth microdilution methods, 2010 to 2012. Journal of clinical microbiology. 2012;50(9):2846-56.
3. Santhanam J, Nazmiah N, Aziz MN. Species distribution and antifungal susceptibility patterns of Candida species: Is low susceptibility to itraconazole a trend in Malaysia? The Medical Journal of Malaysia. 2013;68(4):343-7.
